# Automatic Detection and Classification of Cerebral Microbleeds Using 3D CNN

**DOI:** 10.18178/joig.13.3.275-285

**Published:** 2025-06-12

**Authors:** M. Mohsin Jadoon, Victor Torres-Lopez, Sharjeel A. Butt, Santosh B. Murthy, Guido J. Falcone, Seyedmehdi Payabvash

**Affiliations:** 1 Pak-Austria Fachhochschule: Institute of Applied Sciences and Technology (PAF-IAST), School of Computing Sciences, Pakistan; 2 Department of Electrical Engineering, International Islamic University Islamabad, Pakistan; 3 Department of Neurology, Yale School of Medicine, New Haven, CT, USA; 4 School of Automation, Central South University, Hunan, China; 5 Department of Neurology, Weill Cornell Medicine, New York, NY, USA; 6 Department of Radiology, Columbia University Medical Center, New York, NY, USA

**Keywords:** Cerebral Microbleeds (CMBs), classification, detection, You Only Look Once (YOLO), 3D Convolution Neural Network (CNN)

## Abstract

Cerebral Microbleeds (CMBs) are referred to tiny foci of hemorrhage in brain parenchyma which are smaller than 5 (to 10) mm in size. The presence of CMBs is implicated in pathophysiology of cognitive impairment, dementia, radiation-induced vascular injury, traumatic brain injury, hypertensive microangiopathy, and aging. On brain Magnetic Resonance Imaging (MRI) scans, CMBs appear as hypointense foci, most notable on T2*-weighted or Susceptibility-Weighted Imaging (SWI). Detecting these tiny microbleeds with naked eye is a difficult and time-consuming task for radiologists. In this study we developed an algorithm for automatic detection of CMBs. We applied a two-step strategy: at first, we applied pre-processed 2D image dataset to You Only Look Once (YOLO V2) for detection of CMBs. Then, these detected CMBs locations are used to segment 3D patches from their original SWI volume in the datasets. Next, these patches are used as inputs for Convolution Neural Network (CNN). In the second step, we reduced the number of False Positives (FP) and improved our classification accuracy using 3D CNN. We used two datasets consisting of 979 patients: 879 of whom for training of models, and the remainder for independent validation. We were able to achieve an accuracy of 81% and reduce the $FP_{\mathrm{avg}}$ to 0.16.

## I. Introduction

Cerebral Microbleeds (CMBs) are small foci of hemorrhages that are created by focal accumulations of hemosiderin containing macrophages in brain parenchyma. The paramagnetic properties of these hemorrhagic products lead to susceptibility effects and signal loss on T2*-GRE and Susceptibility-Weighted Imaging (SWI) sequences in brain MRI [1]. The presence of CMBs is associated with higher risk of future intracranial hemorrhage and can be a biomarker for cerebral amyloid angiopathy and cerebrovascular diseases. Recent studies have shown a higher prevalence of CMBs among patients with hematological disorders, brain tumors, abnormalities of blood vessel, hypertension, head trauma, and aneurysm [1]. CMBs are also implicated in pathophysiology of cognitive impairment, and Alzheimer’s dementia [2]. On brain MRI scans, CMBs present as tiny black dots which are best seen on three-dimensional T2*-weighted imaging, SWI, and related techniques [3]. Among different MRI sequences [4], SWI series are the most sensitive technique for identification of CMBs [5].

Currently, brain MRI is the most dependable screening modality for identification of CMBs. Utilization of high-field (3T and higher) magnet MRI scanners and sensitive SWI techniques have improved the sensitivity and accuracy of radiologists in detecting tiny CMBs. In current day-to-day clinical practice, radiologists are tasked to identify CMBs, which implies a subjective and tedious process, prone to human errors. Consequently, CMBs may be missed, ignored, or not consistently reported [6]. Identification of CMBs via Computer Assisted Diagnostic (CAD) appears as a viable option to facilitate, expedite, and increase the accuracy of radiologists in detection and quantification of microbleeds.

## II. Literature Review

So far, many authors have proposed different automated models for identification of CMBs, many of which limited by small sample size [7]. Barnes *et al.* [8] developed an algorithm with statistical thresholding to recognize hypointensities inside the images and utilized Support Vector Machines (SVM) to separate confirmed CMBs. They included 126 CMBs in their dataset and achieved a sensitivity of 81.7%. Bian *et al.* [9] proposed a semi-automatic strategy for recognizing CMBs on SWI series. Their algorithm was based on initial radial symmetry transform to detect CMBs followed by exclusion of FPs by using region growing method. A dataset of 15 patients were used in this study. Fazlollahi *et al.* [10] proposed a two-stage model, using multi-scale Laplacian of Gaussian and Random Forests (RF). Their model was validated on 66 patients and achieved 86 % sensitivity. Chen *et al.* [11] developed an algorithm based on CNN. They used a 20-patient dataset with 117 CMBs and achieved a sensitivity of 89.13%. Wang *et al.* [12] also applied CNN with a rank-based pooling scheme to detect CMBs, and achieved 96.94% sensitivity. However, their dataset only included 10 patients. Hong *et al.* [13] described an algorithm based on CNN using transfer learning and ResNet-50, but also using images from 10 patients. Liu *et al.* [14] also used a CNN-based model in a dataset of 195 patients for training and validation, which was tested on images from 25 patients, achieving 95.8% sensitivity. Chen *et al.* [15] used the 3D residual CNN approach, and reduced the FP average results by 89%. Their dataset consists of 73 patients for training and validation; and 12 patients for testing, achieving 90 % sensitivity. Wang *et al.* [16] described a CMB detection method via 2D-DenseNet Neural Network. They also achieved 97% sensitivity, but using a dataset of 10 patients for training and 10 patients for testing. Al-Masni *et al.* [17] exploited a two-stage strategy, where they adopted YOLO and CNN, consecutively, for the detection of CMBs, and reducing FPs. Their dataset consists of 179 patients, including 107 with low resolution and 72 subjects with high resolution images; and they achieved a sensitivity of 78.85% and 93.62%, respectively. Hong *et al.* [18] utilized sliding neighborhood processing and CNN to detect and classify the microbleeds. They achieved 99% sensitivity but only used the data from 10 patients. Doke *et al.* [19] described the Bayesian optimization to find optimum set of parameters for CNN to detect the CMBs. They also generated data from 10 patients using sliding window operation. Lu *et al.* [20] designed a CNN to identify CMBs. Their model achieved average sensitivity of 98.18% by using a dataset of 20 patients. Tummala *et al.* [21] investigated an ensemble of pretrained Vision Transformer (ViT) models (B/16, B/32, L/16, and L/32) for brain tumor classification using T1-weighted MRI images. The ensemble achieved a high test accuracy of 98.7% on a dataset of 3064 MRI slices, demonstrating the potential of ViT models for aiding radiologists in diagnosis. Hossain *et al.* [22] tackles multiclass brain tumor classification using MRI images, evaluating deep learning models such as VGG16, InceptionV3, ResNet50, and others. The proposed transfer learning model, IVX16, achieved the highest accuracy of 96.94 % on a 3264-image dataset. Explainable AI validated the models, and Vision Transformer (ViT) models were compared for performance. Li *et al.* [23] exploited the ground truth for feature enhancement, and then applied these features for training of a CNN. They achieved an average sensitivity and precision of 90% and 79.7%, respectively. Their dataset included 58 patients, with 50 subjects used for training and testing and 8 subjects used for validation.

**Fig. 1.**
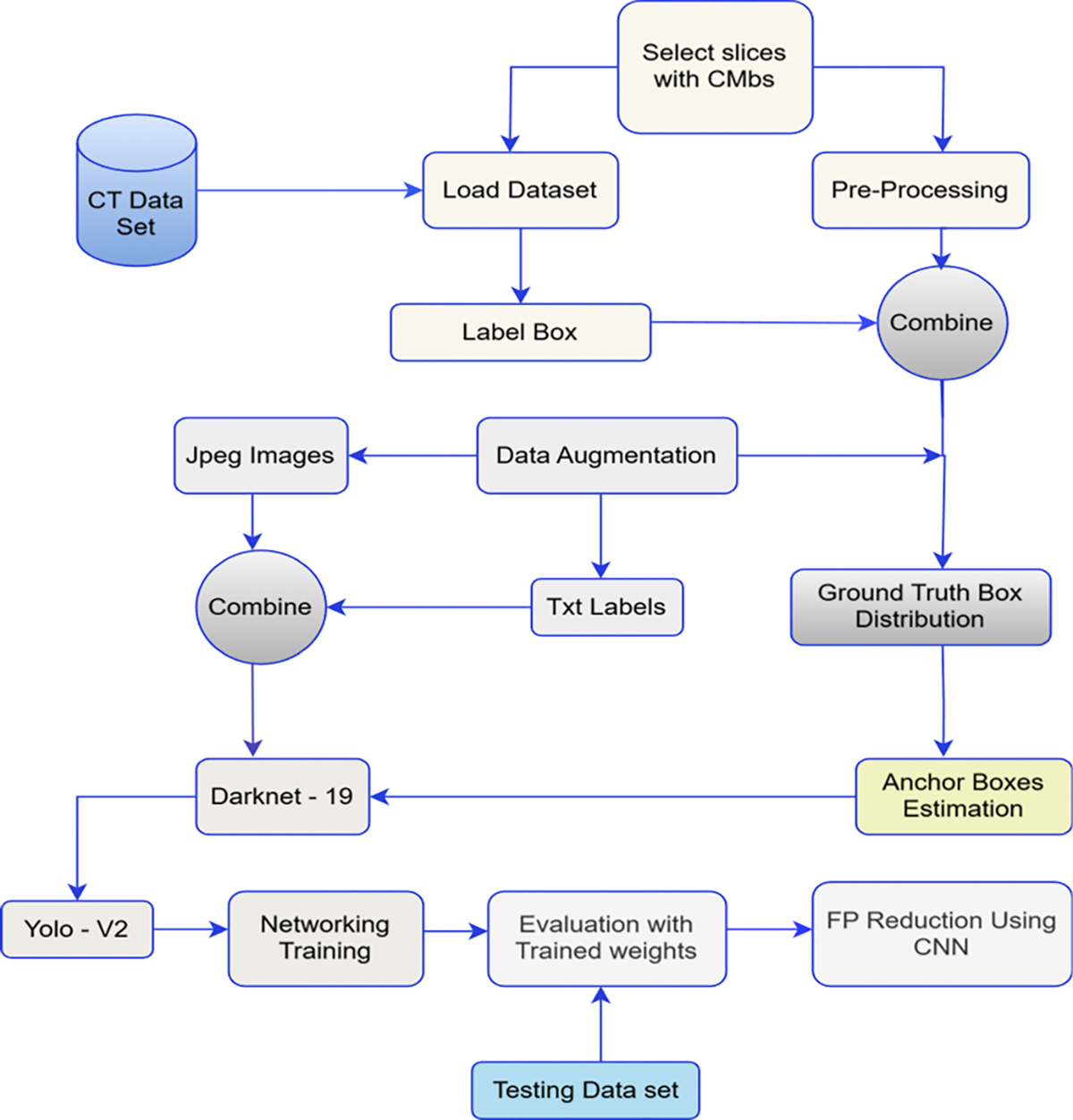
Flowchart of proposed method.

Over the last decade, Artificial Neural Networks (ANN) could achieve extraordinary milestones in the field of computer vision. Consequently, many researchers have deployed ANN models in the field of biomedical imaging. However, the main obstacle in creating generalizable deep learning models for assessment of clinical images is the relatively small size of samples available for training and validation. This is due to the expensive cost of image acquisition and labeling as well as patients’ privacy regulations, which limits public access to medical images. Similarly prior attempts in utilization of deep learning models for detection of CMBs are limited by small sample size in both training and test datasets, as detailed above. Thus, we tried to address this issue by utilizing a large dataset of 979 patients for training and validation of an automated CMB detection model based on YOLO. Fig. 1 describes the flowchart of the proposed method.

The main contributions of this study are:

- Development of a two-step deep learning model for automated CMB detection, utilizing YOLO V2 and Darknet-23 in the first step, followed by a 3D CNN to enhance classification accuracy and reduce False Positives (FPs).
- Training the model on the largest dataset used for CMB detection to date, combining two publicly available datasets, resulting in a total of 879 patients, and testing on 100 patients.

Demonstrating that using a large dataset with preprocessing, augmentation, and fine-tuning significantly improves sensitivity and accuracy while reducing the number of FPs compared to previously reported models.

## III. Materials and Methods

### A. Preprocessing

Image preprocessing is one of the most important steps in Computer Assisted Diagnostic (CAD) systems. As it is often difficult to extract CMBs due to very homogenous transitions in the MR images, therefore in the model training step we applied a contrast enhancement technique to all those slices that has at least one CMB. i.e.,

$$E\overset{\;\text{COLFILT}\;}{\leftarrow }(I,3)$$

where COLFILT filter enhances the image I depending upon the global mean and global variance of the image [24]. To extend the consistency among the input images intensities values, input slices of subjects were normalized in range 0 to 1.

$$E_{\mathrm{norm}\;}=\frac{E_{\mathrm{orig}\;}-E_{max}}{E_{max}-E_{min}}$$

Here $E_{\mathrm{orig}},\;E_{\mathrm{norm}},\;E_{\min}$, and $E_{\max}$ refer to pixel intensities. i.e., original, normalized, minimum and maximum respectively.

### B. Data Augmentation

In deep-learning, models are trained to learn a large number of parameters. This will increase the likelihood of over fitting during training due to the model complexity. Data augmentation can artificially increase the number of subjects to alleviate the risk of over fitting [25]. It artificially creates new sample images by applying transformations such as flipping, rotation and other operations to the actual data sample. For every image, we artificially produced seven new sample images using the combination of 0°, 90°, 180°, and 270° rotations and flipping transformations.

### C. 2D Slices and Brain Extraction

**Fig. 2.**
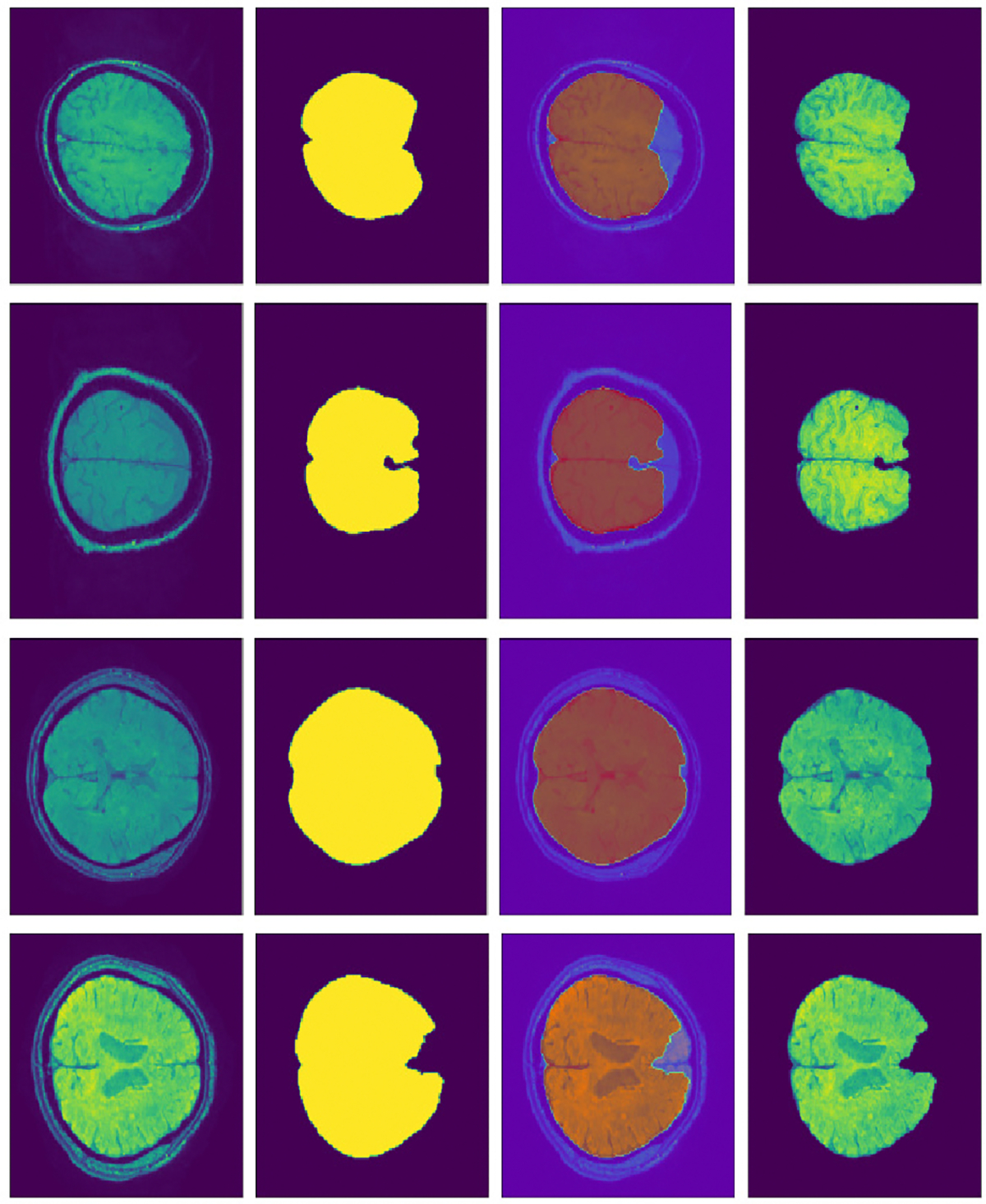
Mask results (left to right) (a) Original Image (b) Mask (c) Fused Image (d) Extracted Image.

In the next step, the augmented data is converted into 2D images with size of 448×448. It created an input matrix $\dot{M}$ of size 6096×448×448. Before applying this input image matrix to YOLO, we also removed the brain skull by apply Brain Extraction Tool (BET) on input data [26]. The UK Biobank dataset, however, provides skull-removed SWI series in for their dataset. Fig. 2 contains the examples of original image, binary mask, fused and brain-extracted image. BET operation produces the output images $\dot{N}$ of size 6096×448×448. Corresponding to each image in $\dot{N}$ we have created a text file. These text files and BET output matrix $\dot{N}$ are given as the input to the YOLO.

### D. YOLO

YOLO is one of the more recent CNN deep learning techniques which is specialized for object identification in images. It can identify areas of interest in images and characterize their classification. Many other CNNs can also (separately) perform the identification and classification tasks but at much higher computational cost. YOLO can simultaneously perform these two tasks in a single convolutional network as a regression problem and with outputs in form of bounding box and class perdition. Indeed, the region-based YOLO strategy has already been applied in medical images, such as detection of lymphocytes on pathological slides [27], identification of lung cancer on low-dose chest CT [28, 29] and characterization of breast abnormalities on mammograms [29]. An overview of YOLO working is given below [30].

Mean square error can be computed after computing the loss function for YOLO. Loss function of training, predicting and target of bounding box is given below [30]:

$${\;\mathrm{Yolo}}_{\mathrm{loss}\;}={\;\mathrm{Localization}}_{\mathrm{loss}\;}+{\;\mathrm{Confidence}}_{\mathrm{loss}\;}\;\;+{\;\mathrm{Classification}}_{\mathrm{loss}\;}$$

where ${\mathrm{Localization}}_{\mathrm{loss}}$ is the error between target and predicted bounding box. ${\mathrm{Localization}}_{\mathrm{loss}}$ coefficient can be calculated as:

$${\mathrm{Localization}}_{\mathrm{loss}\;}=s_{1}\sum_{a=0}^{c^{2}}\;\sum_{b=0}^{d}\;l_{ab}^{obj}\left[{\left(x_{a}-\ddot{x_{a}}\right)}^{2}+{\left(y_{a}-\ddot{y_{a}}\right)}^{2}\right]+\;\;s_{1}\sum_{a=0}^{c^{2}}\;\sum_{b=0}^{d}\;l_{ab}^{obj}\left[{\left(\sqrt{w_{a}}-\sqrt{{\ddot{w}}_{a}}\right)}^{2}+{\left(\sqrt{h_{a}}-\sqrt{{\ddot{h}}_{a}}\right)}^{2}\right]$$

where $s_{1}$ is the weight, c belongs to grid cell and d is the count of bounding box in each c. $\left(x_{a}-{\ddot{x}}_{a}\right)$ represent the center of c and d, (W,h) express the width and height of d in c. $\left({\ddot{x}}_{a},{\ddot{y}}_{a}\right)$ and $\left({\ddot{w}}_{a},{\ddot{h}}_{a}\right)$ are the target’s center in c. If here is an object in d in each c then $l_{ab}^{obj}$ is 1 otherwise it is 0.

${Confidence}_{\mathrm{loss}}$ is the confidence error, if the object is detected in d bounding box of the $C.\;{Confidence}_{\mathrm{loss}}$ is calculated as under:

$$s_{2}\sum_{a=0}^{c^{2}}\;\sum_{b=0}^{d}\;l_{ab}^{obj}{\left(conf_{a}-co\ddot{n}f_{a}\right)}^{2}\;\;+s_{3}\sum_{i=0}^{c^{2}}\;\sum_{j=0}^{d}\;l_{ij}^{obj}{\left(conf_{i}-co\ddot{n}f_{l}\right)}^{2}$$

$s_{2}$ and $s_{3}$ are the weights for the confidence error. $conf_{a}$ express the confidence score of d in c. $co\ddot{n}f_{a}$ is the confidence score of target’s center in c. if there is an object of d in each $c,\;l_{ab}^{obj}$ is 1 otherwise it is 0.

The ${Classification}_{\mathrm{loss}}$ is the error between conditional probabilities of each class in the grid cell a. It is defined in equation below:

$$s_{4}\sum_{a=0}^{c}l_{a}^{obj}\sum_{\alpha \in \text{classes}\;}{\left(P_{a}(\alpha )-{\ddot{P}}_{a}(\alpha )\right)}^{2}$$

Here $s_{4}$, is the weight of Classification error, $P_{a}(\alpha )$ and ${\ddot{P}}_{a}(\alpha )$ ) represents the probabilities of estimated and actual objects class in cell a.

**Fig. 3.**
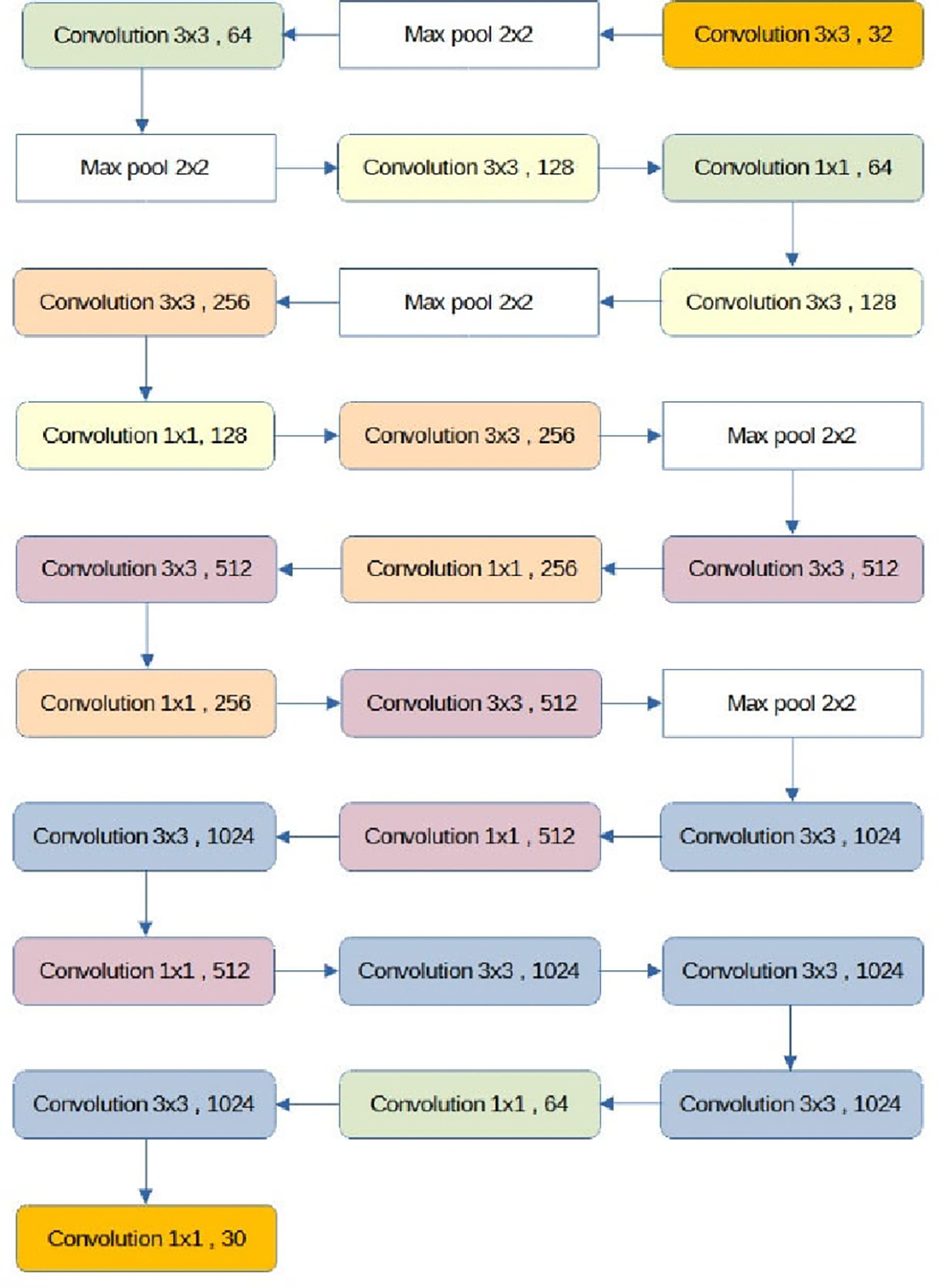
YOLO V2 architecture.

In this study we used YOLO V2 with darknet-23 for the detection of the CMBs. Fig. 3 explains the basic architecture of YOLO V2. This V2 version of YOLO has better detection results and quick execution due to its high network resolution, multi-level training, anchor boxes and batch normalization. For YOLO V2 training, We have used batch size of 16. Momentum and learning rate are 0.9 and 0.0005 respectively.

We applied a two-step strategy: at first, we applied pre-processed 2D image dataset to You Only Look Once (YOLO) for detection of CMBs. Then, these detected CMBs locations are used to segment 3D patches from their original SWI volume in the datasets. Next, these patches are used as inputs for CNN.

### E. 3D CNN

**Fig. 4.**
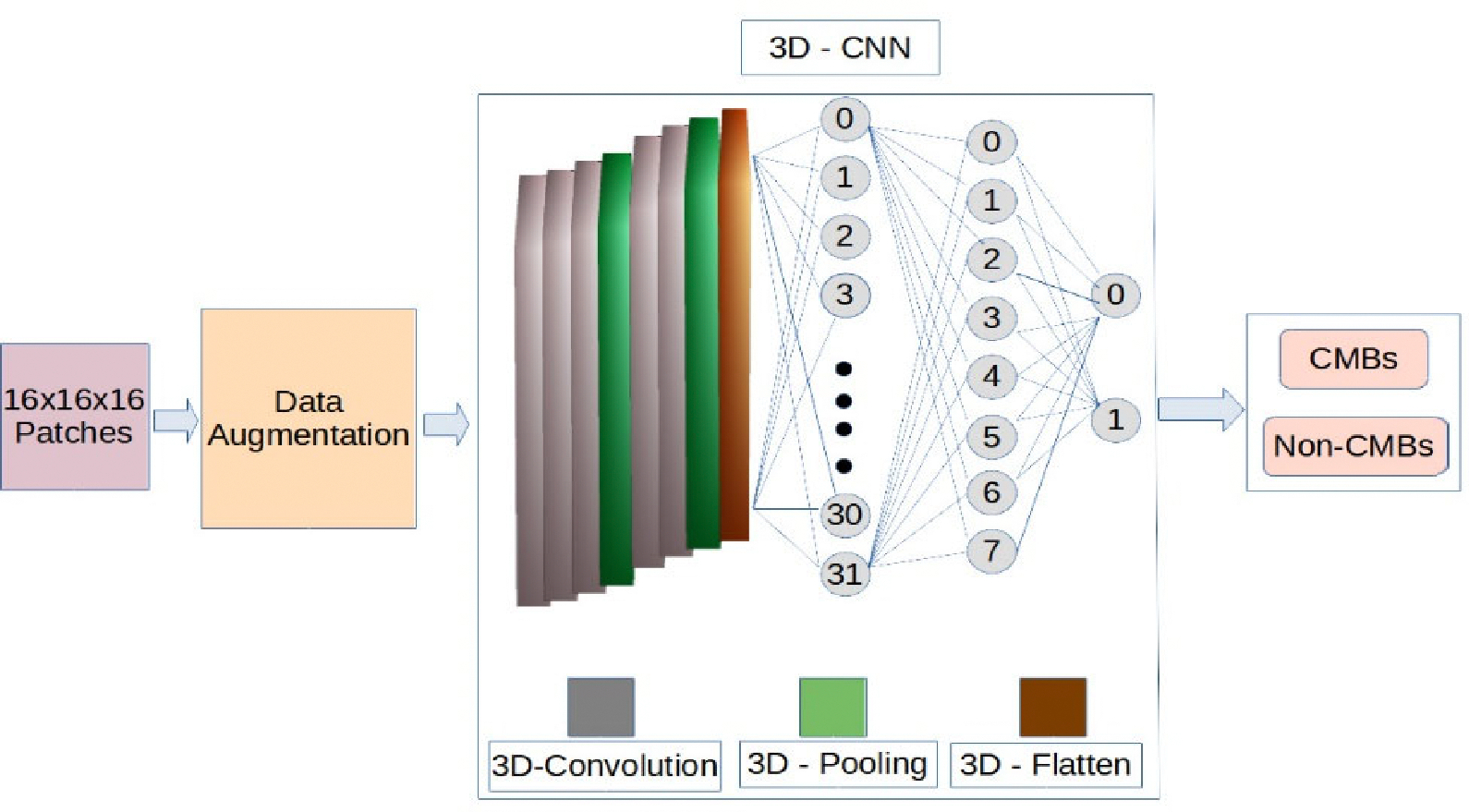
3D CNN Architecture.

The proposed 3D CNN architecture is given in Fig. 4. In 3D convolution layer, set of small kernels is convolved with the feature maps of previous layer. The 3D convolution layer can be formulated as below:

$$w_{i}^{l}(a,b,c)\;=f\left(\sum_{k}\;\sum_{x,y,z}\;w_{k}^{l-1}(a-x,b-y,c\right.\;\;\left.-z)h_{ki}^{l}(x,y,z)+d_{i}^{l}\right)$$

Here, $w_{k}^{l-1}$ represents the $K^{\mathrm{th}}$ feature map of $l-1^{\mathrm{th}}$ layer. $h_{ki}^{l}$ is 3D weights for $w_{i}^{l}$ and $w_{k}^{l-1}$ layers. $d_{i}$ denoted $i^{th}$ biased and f(.) is the activation function.

The activation function used in this model is is ReLU and it is defined as:

$$f(\ddot{x})=ReLU(\ddot{x})=\left\{\begin{array}{cc} x, & \;\mathrm{if}\;\ddot{x}>0\\ 0, & \;\mathrm{if}\;\ddot{x}<0 \end{array}\right.$$

In Eq. (7) the output $w_{i}^{l}(a,b,c)$ represents the feature maps of the current convolution layer. Maximum polling of 2×2 is used for this study and it can be defined as under:

$$\;h_{abc,k}^{l}=max\left(h_{a^{\prime }b^{\prime }c^{\prime },k^{\prime }}^{l-1}\right)\;:a\leq a^{\prime }<a+w,b\leq b^{\prime }<b+h,c\leq c^{\prime }<c+d$$

where, $h_{abc,k}^{l}$ is the output of pooling layer l at abc Position. $h_{a^{\prime }b^{\prime }c^{\prime },k^{\prime }}$ denotes the 3D cube at region $a^{\prime }b^{\prime }c^{\prime }$ in $l-1^{\mathrm{th}}$ layer. Weight, height and depth of pooling layer are represents by w,h,d respectively. Average pooling layer can be defined as given below:

$$h_{abc,k}^{l}=\frac{1}{w\times h\times d}\sum h_{a^{\prime }b^{\prime }c^{\prime }k^{\prime }}^{l-1}\begin{array}{l} a\;\leq a^{\prime }<a+w\\ c\;\leq b^{\prime }<b+h\\ c\;\leq c^{\prime }<c+d \end{array}$$

A flatten layer is used to convert the multidimensional input of previous layer into the one dimensional data. Optimizers are used to calculate and update the network parameters that affect model training and its output. The Adam optimizer have the advantages over other optimizers. It combines the characteristics of AdaGrad and RMSProp optimizer and has high efficiency, convenient implementation and its parameters are updated without gradient transformation. Therefore, for the 3D CNN model we used the Adam optimizer. Adam’s update steps are given bellow. To calculate the exponential moving average of the gradient and $T_{0}$ is initialized to 0.

$$g_{t}=\nabla_{\theta }J\left(\theta_{t-1}\right),$$

$$T_{t}=\alpha_{1}T_{t-1}+\left(1-\alpha_{1}\right)g_{t}$$

Then, calculate the exponential moving average of the square of the gradient; $v_{0}$ is initialized to 0.

$$v_{t}=\alpha_{2}v_{t-1}+\left(1-\alpha_{2}\right)g_{t}^{2}$$

The deviation correction is performed on the gradient mean $T_{t}$ and the gradient variance $v_{t}$.

$${\overset{ˆ}{T}}_{t}=\frac{T_{t}}{\left(1\;-\;\alpha_{1}^{t}\right)}$$

$${\overset{ˆ}{v}}_{t}=\frac{v_{t}}{\left(1\;-\;\alpha_{2}^{t}\right)}$$

To update the parameters, the initial learning rate γ is multiplied by the ratio of the gradient mean to the square root of the gradient variance.

$$\Theta_{t}=\theta_{t-1}-\gamma^{*}\frac{{\hat{T}}_{t}}{\sqrt{{\overset{ˆ}{v}}_{t}}\;+\;\delta }$$

In above equations, $\alpha_{1}$ represents the exponential decay rate, which controls the weight assignment, usually taking a value close to 1, with a default of 0.9; $\alpha_{2}$ represents the exponential decay rate, which weights the mean of the gradient squares, with a default of 0.99; $\delta =10^{-7}$, which prevents the denominator from being 0.

**Fig. 5.**
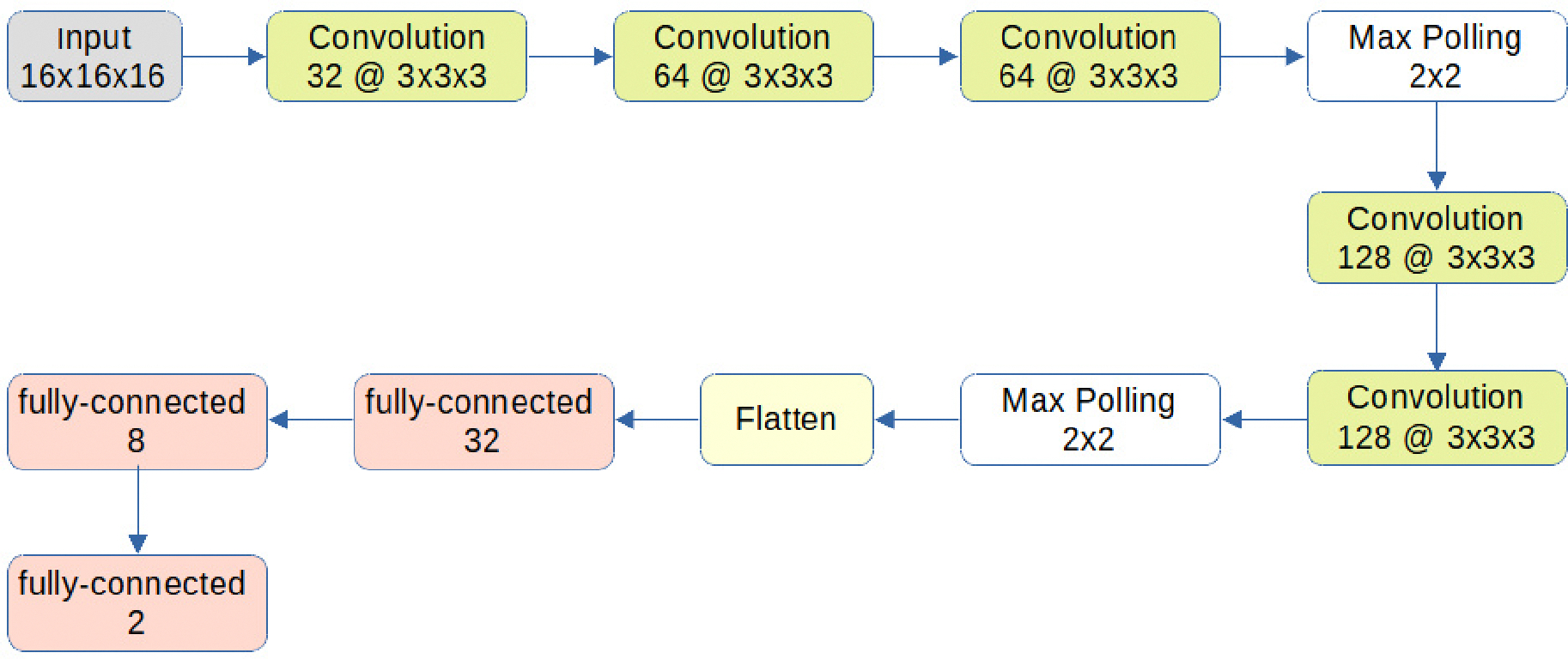
3D CNN layers detail.

Detailed framework of proposed architecture is illustrated in Fig. 5. The five convolution and two max pooling layers are used. Convolution layers have kernels with size 3×3×3 and pooling layers are with kernels of size of 2×2. The dropout ratio used at this stage is 0.4. Whereas, the learning rate is 1×10^−7^ and batch size is 50.

## IV. Datasets

For this study, we utilized two publicly available datasets. The first dataset was from Gachon University Gil Medical Center (GUGMC) [17], and the second dataset was from UK Biobank [31].

### A. Original Dataset

The MRIs in GUGMC dataset were obtained utilizing 3.0 T Verio and Skyra Siemens MRI scanners. It has a total of 179 patients, out of whom, 72 patients had 188 microbleeds with an image matrix size of 512×448×72. The remaining 107 patients had 572 microbleeds and an image matrix of size 288×252×72. We also included 800 patients from the multicentric UK Biobank dataset with an image matrix size of 288×256×48 (https://www.ukbiobank.ac.uk/enable-your-research/about-our-data/imaging-data). All of GUGMC dataset in addition to 100 patients from the UK Biobank were used to train the YOLO model for detection of CMBs.

In FP reduction part, we used all the GUGMC data and 700 patients from UK Biobank to train the 3D-CNN model. For the FP reduction, detected CMBs locations from YOLO were used to segment 16×16×16 patches from their respective images in the dataset. Thus, the size of input image for the 3D-CNN model was set as 16×16×16. Again augmentation operation were applied to these segmented patches. For every image, we artificially produced seven new sample images using the combination of 0°, 90°, 180° and 270° rotations and flipping transformations. Moreover, these rotations and flipping were across each axis. Thus, the resulting dataset contained twenty-two times more images than the original series. The remaining 100 patients from the UK Biobank were used for independent testing of the proposed models.

### B. Ground Truth Labeling

The ground truth labels for GUGMC dataset were available online. For UK Biobank dataset, a neuroradiologist (SP)—with 11 years of experience in interpretation of brain MRIs – reviewed and generated the ground truth labeling for the presence of CMBs. The labeling of ground truth was performed as per international standards [32]. The diameter of CMB were set as ≤ 10 mm.

### C. Training and Testing

In order to determine the generalizability and reliability of our proposed model, we applied K fold cross-validation, setting the value of K = 5. Both datasets were split into five folds separately, first fold of each dataset were used for validation while other four for training and testing purpose. The proposed research was performed on the clusters of Yale university, with 4 CPU per Node and 3 GPU of NVIDIA-SMI 450.80.02. The CMBs detection and their classification were performed by the Python programming language using keras and tensorflow.

## V. Result and Discussion

In this section, results of the proposed algorithms are presented. As we have discussed in literature review section that many studies have adopted two stages strategy for detection of CMBs due to large number of FP in single-stage models. Our method performs well in both detection and FP reduction stages. At Stage 1, it has already achieved very low $FP_{\mathrm{avg}}$ and 100% sensitivity even on large test dataset. All other method at Stage 1 neither could able to achieve as low $FP_{\mathrm{avg}}$ and nor achieved as high sensitivity and they are missing the patients even in low test dataset.

Although, at Stage 1 our model already has achieved the $FP_{\mathrm{avg}}$ of 0.37 that other models not able to achieved even after two stages. The proposed model after utilization of a large dataset for training with appropriate prepossessing, augmentation and fine tuning of the deep learning model have provided higher sensitivity and accuracy as well as lower number of FPs.

### A. Evaluation Metrics

The proposed method is evaluated in terms of True Positive (TP), False Positive (FP) patients, specificity, precision, sensitivity, and False Positive average $\left(FP_{\mathrm{avg}}\right)$.

In medical image classification, a False Positive (FP) is the incorrect classification of subjects, i.e., the model predicts the presence of disease while in reality the subject is disease-negative. On the other hand, a False Negative (FN) is the incorrect classification of subjects where a test result incorrectly indicates the absence of a disease. A TP is the correct classification of positive subjects, whereas a true negative is the correct classification of negative subjects. Specificity is the most commonly used assessment measure, and it represents all the negative cases with TN or FP.

$$\mathrm{Specificity}\;=\frac{TN}{FP\;+\;TN}$$

Precision or Positive Predicated Value (PPV) is defined as the number of correctly detected positive cases over all detected positive cases.

$$PPV=\frac{TP}{FP\;+\;TP}$$

Sensitivity is defined as the proportion of the detected positive cases over the actual positive cases, including only disease-positive subjects.

$$\mathrm{Sensitivity}\;=\frac{TP}{FN\;+\;TP}$$

Accuracy (Acu) is sum of the total number of true values in test dataset and is defined as below:

$$\mathrm{Accuracy}\;=\frac{TP\;+\;TN}{TP\;+\;TN\;+\;FP\;+\;FN}$$

The False Positive Average ($FP_{\mathrm{avg}}$) is number of false positive per subject and is defined as:

$$FP_{avg}=\frac{FP}{K}$$

where K represents the number of subjects in testing dataset.

The Matthews Correlation Coefficient (MCC) is used in machine learning as a measure of binary classifications. The coefficient takes into account all the true and false values either they are positives or negatives and is regarded as a balanced measure which can be used even if the classes are of very different sizes. It returns a value between −1 and +1.

$$MCC=\frac{TP\;\times \;TN\;-\;FP\;\times \;FN}{\sqrt{\left(TP\;+\;FP)(TP\;+\;FN)(TN\;+\;FP)(TN\;+\;FN\right)}}$$

### B. Detection of CMBs

**Fig. 6.**
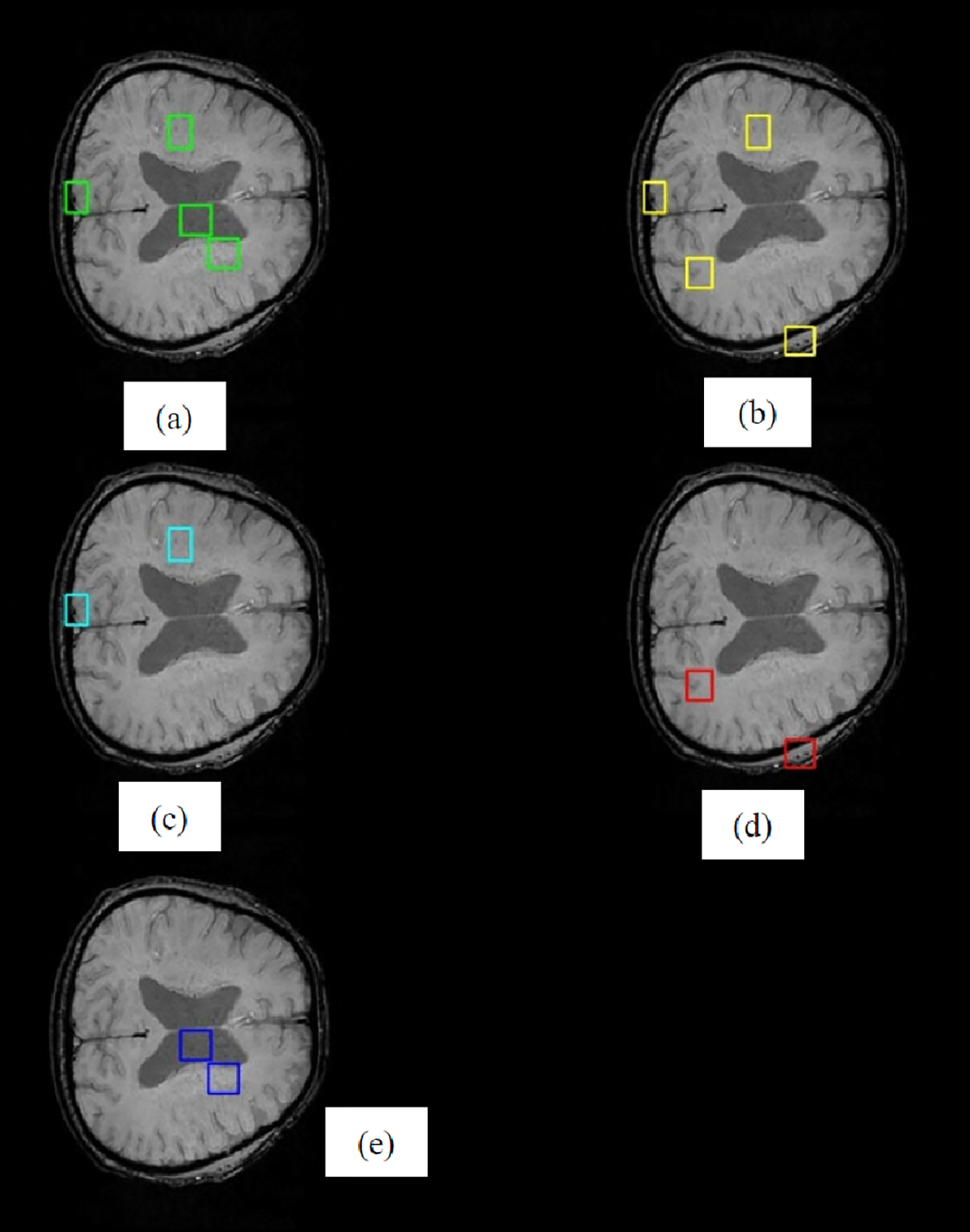
Detection Results (a) True Labels; (b) Predicted Labels; (c) True Positives; (d) False Positives; (e) False Negatives.

First, we trained the model using the GUGMC dataset. This dataset had the 762 CMBs therefore, we gave notation to our trained model as $T_{762}$. Fig. 6 presents the detection results, including (a) the ground truth labels, (b) the predicted labels, (c) the correctly identified instances (true positives), (d) the incorrectly identified instances (false positives), and (e) the missed detections (false negatives). Table I represents the results of this experiment on validation dataset. With Confidence Score (CS) of 0.5, out of 14 patients 12 of them are detected successfully and 2 patients are detected as FN, the number of FP are 62. Then we evaluated the results with CS of 0.6, where we could only identify 5 patients as TP. There are 9 FN, while 14 are detected as FPs. Given the number of FPs, we trained the model with another 100 patients from UK Biobank. These 100 patients had 102 CMBs so we denoted the model as $T_{864}$. Again, applying 5-fold cross validation to add additional data in each fold.

Table II describes the results on validation dataset, with 0.5 and 0.6 CS. Here, all 20 patients with CMBs are detected correctly and 37 patients are detected as FP, with sensitivity, specificity, and accuracy of 1, 0.65 and 0.71, respectively. Similarly, with CS of 0.6, model detected 11 patients as TP. There are 15 FP but number of FN increased up to 9.

Thus, the results of the $T_{864}$ model with SC 0.5 were most promising: no FN, and reduced FP rate of 37 in validation dataset. To further improve our model, we trained our model with another set of 100 patients from the UK Biobank. These additional patients had 24 CMBs so we denoted the model as $T_{888}$. Again, applying 5-fold cross validation, Table III summarizes the results of the model on validation dataset, with 0.5 and 0.6 CS. Model $T_{888}$ successfully detected all patients with CMBs. However, there was an increase in the FP rate. In this model, the CS of 0.6 led to a high rate of FN as well.

As per results summarized in Tables I–III, $T_{864}$ with CS of 0.5 appeared to have the optimal predictions among all models. In Table IV, we present the prediction results in 5 test fold in cross-validation of model $T_{864}$ with 0.5 of CS. In all folds, the models achieved high sensitivity but with high FP rate.

Then, we tested our models on an isolated cohort of 100 patients (Table V). Model $T_{762}$ detects 12 TP with 2 FN, while there are 24 TN and 62 FP. Model $T_{864}$ had better performance, as there is no FN and FP rate is reduced to 37. Model $T_{888}$, however, only identified 1 TN but with 54 FP.

We also tested our model, applying CS of 0.55, on an isolated cohort of 100 patients (Table VI). Out of 14 patients with CMBs, model $T_{762}$ detects 8 TP with 36 FP. Again, model $T_{864}$ had better performance, but still with 3 FN and 25 FP. Model $T_{888}$ achieved almost same results as $T_{864}$ but with 37 FP.

Table VII summarizes the results of CMB detection applying CS of 0.6 in different models on the 100-patient test dataset. For CMB detection, applying a CS of 0.6 reduced the sensitivity of all models, while increasing their specificity (mostly as a result of the drop in number of FP subjects). With CS of 0.6, low TP rate was the major issue for performance of all models, with model $T_{864}$ again outperforming others.

### C. False Positive Reduction

As depicted in Tables I–VII, all models had relatively high rate of FP in their predictions. To mitigate the high FP rate, we devised a 3D CNN model to improve the classification accuracy.

Table VIII depicts the prediction results from applying 3D CNN on output of YOLO-based models with CS of 0.5. Here, the output of each model ($T_{762},\;T_{864}$ and $T_{888}$) was separately given as input to 3D CNN model for binary classification. Model $T_{762}$ predicted 8 TP and 45 TN, it has sensitivity of 0.57 and $FP_{\mathrm{avg}}$ dropped to 0.41. Model $T_{888}$ predicted 11 TP and 54 TN, it has sensitivity of 0.78 $FP_{\mathrm{avg}}$ and reduced to 0.32. Model $T_{864}$ produced the best result with 11 TP and 70 TN. There are 3 FN, with $FP_{\mathrm{avg}}$ reduced to 0.16. The model achieved an accuracy of 0.81 with sensitivity and specified of 0.78 and 0.81, respectively.

We also tested the results of model after applying CS of 0.55 in YOLO prediction models. Table IX shows that 3D CNN could reduce the FP rate in output of YOLO-based models using CS of 0.55. Model $T_{762}$ predicted 6 TP and 64 TN, with sensitivity of 0.42 and $FP_{\mathrm{avg}}$ of 0.22. Model $T_{864}$ predicted 8 TP and 73 TN, achieving specificity of 0.84 with $FP_{\mathrm{avg}}$ reduced to 0.13. However, there is a drop in sensitivity to 0.57. Similarly, the 2nd step 3D CNN reduced the $FP_{\mathrm{avg}}$ to 0.18 for model $T_{888}$ output, but with a concomitant drop in sensitivity to 0.64.

Table X shows the results from reduction of FP rate after applying 3D CNN on output of YOLO-based models with 0.6 CS threshold. All models achieved >0.8 accuracy and reduced the $FP_{\mathrm{avg}}$ to <0.1; however, there is a drop in prediction sensitivity. Models $T_{762},\;T_{864}$ and $T_{888}$ achieved sensitivity of 0.28, 0.57, and 0.50 respectively.

In summary, model $T_{864}$ with CS of 0.5 had the best performance after 3D CNN reduction of FP subjects. In Table XI, we present the results of 5 fold test for false positive reduction in model $T_{864}$. The proposed model was trained on 700 patients dataset. All the CMBs were split into 5 folds. Each time 4 folds were used as training, while 5th fold was used for the validation only. In all 5 fold, model has performed achieved >0.7 sensitivity, >0.8 specificity, and >0.8 Accuracy with lower FP rate compared to the first-step YOLO-based models. Table XII shows the comparison of existing state of art techniques with our proposed method. Our proposed model outperforms the existing techniques, as it has tested on large dataset and able to reduces the $FP_{\mathrm{avg}}$ to the significant level.

## VI. Discussion

This study proposes a new two-step model for automated detection of CMBs, that is highly generalizable and outperformes previously reported models in terms of sensitivity, accuracy and $FP_{\mathrm{avg}}$. The accurate detection of CMBs is of interest for a plethora of different diseases. The prior research indicates a role of CMBs as diagnostic and prognostic markers for cerebrovascular disease [34]. While CMBs are also found in healthy populations, where prevalence rises strongly with increasing age, they can also be indicative of an underlying small vessel disease [35]. The spatial distribution of the CMBs tends to differ between different causes of small vessel disease, with cerebral amyloid angiopathy most frequently causing lobar CMBs, while classic cerebrovascular risk factors such as hypertension tend to cause deep CMBs [36]. Lobar CMBs were shown to have a high positive predictive value for cerebral amyloid angiopathy, even in patients without manifest Lobar Intracerebral Hemorrhage (ICH) [37]. This suggests that, given correct detection and localization, CMBs could helpful to infer the nature of a present small vessel disease. The overall presence and location of CMBs can also be used as a marker of cerebrovascular risk. The risk of ischemic stroke as well as ICH is increased in stroke-free individuals given the presence of CMBs [38]. CMBs can also play a role when predicting the risk of repeated hemorrhage in patients that initially present with a hemorrhagic stroke [39].

The role of CMBs in cognitive impairment and dementia have also been subject of interest over the past two decades. While the exact causality between loss of cognition and presence of CMBs has not been fully understood yet, two (non-exclusive) hypothesis are commonly discussed: CMBs could have a direct effect onto cognition by disrupting the cerebral network [40], or they could be a manifestation of the underlying brain pathology, which in turn causes the deficits. The Rotterdam study found an association between presence of microbleeds and decreased cognitive function in patients without dementia, even after adjustment for vascular risk factors and other imaging markers of small vessel disease. Future research on the clinical significance of CMBs as marker of cerebrovascular risk, Alzheimer disease and other areas of interest would greatly benefit from a more sensitive and accurate detection of CMBs.

In this study, we perform different pre-possessing operations, including image enhancement using COLFIT filter is used to enhance the Region of Interest (ROI), image normalization is used to increase the consistency among the input intensities. Moreover, skull stripping is a major phase in MRI brain imaging applications and it refers to the removal of the brain’s noncerebral tissues. The main problem in skull-stripping is the segmentation of the non-cerebral and the intracranial tissues due to their homogeneity intensities. In this study, Skull removal was accurately perform using BET. To avoid the over fitting during training due to the model complexity, we used data augmentation. We artificially creates new sample images by applying transformations such as flipping, rotation and other operations to the actual data sample. For CMBs detection we have used YOLO model, finding an optimal model for detection is a challenging problem as the function may have multiple parameters inputs need to be tuned. After several hours long training’s and extensive experimental trial optimal selection of model is made. Our proposed model achieved outstanding results at 1st stage. Similarly, for FP reduction a 3D CNN model is proposed, again due to optimal model, architecture layers selection and fine tuning of parameters enable our proposed model to achieved the lowest $FP_{\mathrm{avg}}$ rate as compare to other existing models. For medical applications, another main hurdle is creating generalize deep learning models. This is due to the relatively small size of samples available for training and validation. To generalize the results we have used the largest dataset for training and validation for an automated CMB detection model.

## VII. Conclusion

New generation of MRI scanners and sequences facilitate depiction of tiny CMBs particularly on SWI series. While CMBs are implicated in several neurological disorders, their identification and reporting in routine clinical practice impose a tedious process for radiologists and is prone to human errors. In this study, we proposed a two-step automated detection algorithm for localization and classification of CMBs. In the first step, we localized CMBs using YOLO V2, which achieved high sensitivity but with relatively high FP rate. Then we used the output of YOLO-based model as an input for a 3D CNN for further improving the classification accuracy and reducing the FP rates. After the FP reduction stage, our proposed model FP rate dropped to 0.16 with 0.81 accuracy, 0.81 specificity, and 0.78 sensitivity. The proposed algorithm can be used for automated detection of CMBs in imaging mega-data to investigate the neurobiological consequences of CMBs in different disease entities.

## Figures and Tables

**TABLE I.** Detection of CMBs with $T_{762}$ on Validation Dataset

Trained CMBs	Conf score = 0.5			Conf score = 0.6		
		
$T_{762}$	**TP**	**FN**	**FP**	**TP**	**FN**	**FP**
	12	2	62	5	9	14

**TABLE II.** Detection of CMBs with $T_{864}$ on Validation Dataset

Trained CMBs	Conf score = 0.5						Conf score = 0.6					
		
$T_{864}$	TP	FN	FP	Sen	Spe	Acu	TP	FN	FP	Sen	Sep	Acu
	20	0	37	1	0.65	0.71	11	9	15	0.55	0.83	0.77

**TABLE III.** Detection of CMBs with $T_{888}$ on Validation Dataset

Trained CMBs	Conf score = 0.5			Conf score = 0.6		
		
$T_{888}$	TP	FN	FP	TP	FN	FP
	11	0	54	8	6	17

**TABLE IV.** Fold Test of model $T_{864}$ with Conf Score = 0.5

FOLD	TP	FN	FP	TN	Sen	Spe	Acu

Fold 1	11	2	32	61	0.84	0.65	0.67
Fold 2	20	0	37	71	1	0.65	0.71
Fold 3	12	2	37	70	0.85	0.64	0.66
Fold 4	17	0	39	47	1	0.54	0.62
Fold 5	15	1	28	55	0.93	0.66	0.7

**TABLE V.** Detection of CMBs with Confidence Score = 0.5 on 100 Patients

Trained CMBs	TP	FN	FP	TN	Sen	Spe	Acu

$T_{762}$	12	2	62	24	0.85	0.27	0.36
$T_{864}$	14	0	37	49	1	0.56	0.63
$T_{888}$	13	1	54	32	0.92	0.37	0.45

**TABLE VI.** Detection of CMBs with Confidence Score = 0.55 on 100 Patients

Trained CMBs	TP	FN	FP	TN	Sen	Spe	Acu

$T_{762}$	8	6	36	50	0.57	0.58	0.58
$T_{864}$	11	3	25	61	0.78	0.7	0.72
$T_{888}$	11	3	37	49	0.78	0.56	0.6

**TABLE VII.** Detection of CMBs with Confidence Score = 0.6 on 100 Patients

Trained CMBs	TP	FN	FP	TN	Sen	Spe	Acu

$T_{762}$	6	8	14	72	0.42	0.83	0.78
$T_{864}$	10	4	12	74	0.71	0.86	0.84
$T_{888}$	8	6	17	69	0.57	0.8	0.77

**TABLE VIII.** Reduction of False Positive CMBs with 3D CNN, Confidence Score = 0.5 on 100 Patients

Trained CMBs	TP	FN	TN	Sen	Spe	MCC	Acu	$FP_{\mathrm{avg}}$

$T_{762}$	8	6	45	0.57	0.52	0.06	0.53	0.41
$T_{864}$	11	3	70	0.78	0.81	0.46	0.81	0.16
$T_{888}$	11	3	54	0.78	0.62	0.28	0.65	0.32

**TABLE IX.** Reduction of False Positive CMBs with 3D CNN, confidence score = 0.55 on 100 Patients

Trained CMBs	TP	FN	TN	Sen	Spe	MCC	Acu	$FP_{\mathrm{avg}}$

$T_{762}$	6	8	64	0.42	0.74	0.13	0.7	0.22
$T_{864}$	8	6	73	0.57	0.84	0.35	0.81	0.13
$T_{888}$	9	5	68	0.64	0.79	0.33	0.77	0.18

**TABLE X.** Reduction of False Positive CMBs with 3D CNN, confidence score = 0.6 on 100 Patients

Trained CMBs	TP	FN	TN	Sen	Spe	MCC	Acu	$FP_{\mathrm{avg}}$

$T_{762}$	4	10	80	0.28	0.93	0.24	0.84	0.06
$T_{864}$	8	6	78	0.57	0.9	0.34	0.86	0.08
$T_{888}$	7	7	78	0.5	0.9	0.39	0.85	0.08

**TABLE XI.** 5 Fold Test of our model for False Positive Reduction Trained on 700, Conf score = 0.5

FOLD	TP	FN	FP	TN	Sen	Spe	Acu

Fold 1	13	2	16	69	0.86	0.81	0.82
Fold 2	18	2	9	71	0.9	0.88	0.89
Fold 3	11	0	12	77	1	0.86	0.88
Fold 4	6	2	4	88	0.75	0.95	0.94
Fold 5	5	2	4	89	0.71	0.95	0.94

**TABLE XII.** Comparison of Existing Techniques with Our Proposed Method

Method	Total/Test Patients	Sensitivity	Precision	$FP_{\mathrm{avg}}$

2D-ResNet-50 [13]	10/-	95.71	99.18	3.4
1st stage: 3D-FRST [14]	179/41	99.4	-	276.8
2nd stage: 3D-ResNet		95.24	70.9	1.6
1st stage: 2D-FRST [15]	61/12	86.5	-	231.88
2nd stage: 3D-ResNet		94.69	71.98	11.58
2D-DenseNet [16]	20/-	97.78	97.65	11.8
1st stage: YOLO [17]	151/37	93.62	-	52.18
2nd stage: 3D-CNN		94.32	61.94	1.42
1st stage: 3D-FCN [33]	106/20	98.29	-	282.8
2nd stage: 3D-CNN		93.16	44.31	2.74
Proposed 1st stage: Yolo V2 with $T_{864}$	279/100	1	-	0.37
Proposed 2nd stage: 3D-CNN CNN with $T_{864}$	879/100	78.57	40.7	0.16
